# Supplementary material for: Cloud BioLinux: pre-configured and on-demand bioinformatics computing for the genomics community
Source: BMC Bioinformatics. 2012 Mar 19;13:42. doi: 10.1186/1471-2105-13-42 (PMC3372431; doi:10.1186/1471-2105-13-42)
Supplement: Additional file 1 — Supplementary 1 Cloud BioLinux software documentation in the form of a mini, self-contained website. Users need to download and uncompress the .zip file, and open through a web browser the "index.html" file available on the main directory. (ZIP 1823 kb). [file 1471-2105-13-42-S1.ZIP › Cloud-BioLinux-Package-Documentation/docs/readseq.html]

Bio-Linux Software Documentation Pages

Back to search form

## readseq

|  |  |
| --- | --- |
| Name | readseq |
| Description | **Readseq** allows the user to read & reformat biosequences  **Readseq** was written originally around 1989 as a component of a sequence analysis program, but when a small, simple command-line interface was added, it took on a life of its own as a conversion program for bioinformatics. It's main contribution to bioinformatics is that it takes on the job of guessing what your input biosequence data format is, and converting it to what your software knows how to handle. |
| Homepage | http://iubio.bio.indiana.edu/soft/molbio/readseq/java/ |
| Remote Documentation | http://iubio.bio.indiana.edu/soft/molbio/readseq/java/readseq-help.html |
